# Are “part-time” general practitioners workforce idlers or committed professionals?

**DOI:** 10.1186/1471-2296-15-154

**Published:** 2014-09-19

**Authors:** Kathryn M Dwan, Kirsty A Douglas, Laura E Forrest

**Affiliations:** Australian National University, Canberra, Australia; Peter MacCallum Cancer Centre, Melbourne, VIC Australia

**Keywords:** General practitioners, General practice, Health workforce, Quality patient care, Part-time

## Abstract

**Background:**

The traditional view of general practice holds that only general practitioners (GPs) in full-time clinical practice can provide quality patient care. Nevertheless, increasing numbers of GPs are choosing to work sessionally, that is, ostensibly “part-time”. There are concerns about the health workforce’s ability to meet demand and also fears that patient care may be compromised. We sought answers to a) what activities do GPs undertake when not consulting patients, b) why do they choose to work sessionally, and c) does sessional general practice reflect a lack of commitment to patients and the profession?

**Methods:**

Semi-structured interviews were conducted with GPs who worked sessionally, (i.e. six or fewer sessions a week in clinical general practice, where a session comprises four consecutive hours of patient care). These data were analysed qualitatively and saturation was reached.

**Results:**

The majority of participants were in full-time paid employment, while part-time in clinical general practice. They reported that consultations increasingly required the management of patients with complex, chronic conditions who also required psychological management. Coupled with unrealistic patient expectations, these factors led GPs to be concerned about maintaining the quality patient care they considered professionally desirable. Many diversified their work activities to ensure that they retained their professional standards.

**Conclusion:**

“Part-time” general practice is a misnomer that masks the contribution these GPs make as part of the health workforce. Sessional practice more accurately describes the nature of our participants’ clinical work. Their choice of sessional work is a professional response to the increasing demands within the consultation. It enables GPs to maintain their commitment to quality patient care and their profession, while attenuating the challenges of demanding consultations. Sessional general practitioners demonstrate strong commitment to their patients and the profession.

## Background

Health workforce shortages may be felt most keenly by developing nations [1, 2], but are a concern for all [3]. Developed nations are particularly worried about the number of general practitioners (GPs) available to service their ageing populations [4–10]. Foremost among the factors believed to contribute to GP shortages is the tendency for female and younger GPs to work less than full-time in patient care [11–13]. The medical press, at least in Australia, plays a role in promulgating the view that a less than full-time patient load reflects a lack of commitment to patients and the profession [14–16], but is this so?

Women are indeed more likely to choose general practice and to work sessionally (i.e. less than a full-time clinical load) because of caring commitments [17–21]. However, male doctors are beginning to make similar choices, with the hours worked per week by both genders declining [22, 23], and the presence of young children in the family predicting male preferences [24]. Some British research suggests that “the new general practice” distinguishes itself from “traditional” general practice in the way that quality of work and life has replaced commitment to patients and the profession [25], but there is no evidence that explicitly links Generation Y with workforce shortages [13].

Ever decreasing rates of full-time clinical practice need to be factored into workforce planning, given the likelihood that women will continue to make family friendly choices, and that a healthy work-life balance for both genders is something to be encouraged [26–28]. However, workforce planning requires an understanding of all aspects of the workforce’s behaviour and preferences. While many studies have observed a rise in sessional practice, they rarely queried whether this represented their subjects’ only professional occupation. We were prompted to look at this issue differently and our starting point was to establish what GPs do when not consulting with patients.

In 2008 Australia’s capital had just two thirds the desired level of GPs [29], even though there were sufficient registered to service the population. A working group – comprising national and local health officials as well as representatives from three medical organisations – was convened to grapple with this issue. The choice to work less than full-time in clinical practice appeared to account for the shortfall, in part. The authors were commissioned to investigate the nature and extent of GPs’ paid and unpaid work, why some choose to work less than full-time, and whether sessional work reflects a lack of commitment to patients and the profession.

## Methods

### Definitions

General practice was defined as seeing un-referred patients within a primary care setting. Subspecialties, such as maternal and child health, drug and alcohol, or Indigenous health, were excluded because the Royal Australian College of General Practitioners specifies that GPs care “for patients of all ages, both sexes, children and adults across all disease categories” and “over a period of their lifetime” [30]. Sessional, or part-time, general practice was defined as six or fewer sessions a week where a session comprises four consecutive hours of patient contact.

### Subjects

GPs who reported that they worked six or fewer clinical sessions in general practice in the 2007 Australian Capital Territory (ACT) GP Census were invited to participate. Twelve GPs who met the predetermined criteria contacted the research team and were interviewed. Personal connections and word of mouth were used to purposively sample additional GPs, ensuring a range of ages, experiences and gender [31].

### Ethics

Ethical clearance was granted by both the ACT Health and the Australian National University (ANU) Human Research Ethics Committees and all participants gave written consent [31].

### Design and analysis

We were interested in the how participants’ managed their lives and how they viewed their work as sessional GPs, as well as the determinants and consequences of those views [32, 33]. Data was collected via semi-structured interviews. The interview schedule was developed using Wengraf’s algorithm for designing interviews and analysing data, which entails linking theoretical concepts to possible empirical indicators [34]. In discussion with the working group and with reference to the literature we identified the central research questions and developed the specific interview questions. The schedule was shortened slightly after the conduct of a pilot interview.

The interviews occurred between October 2008 and February 2009. Participants were assigned unique identifiers that included their gender and their age in decades. All interviews were recorded and transcribed verbatim, and both lead researchers checked all transcripts for accuracy. NVivo9 was used to facilitate coding. Data saturation was achieved [35]. Initial coding was based around the central research questions and had a tree like structure. Additional categories were added as new themes emerged.

Several strategies were used to validate the findings. The research team consisted of one GP in sessional practice and two social scientists. This arrangement limited the possible influence the GP may have brought to bear on the analysis and interpretation. One social scientist coded all the interviews and the remaining two authors then coded the interviews using the same coding tree. Coding was compared for consistency and disagreement was resolved via discussion among authors. Data was deliberately searched for instances that expressed views contrary to prevailing themes; none were found. The initial findings were tested and verified at a meeting that included representatives from the working group and two research participants.

## Results

### The sample

Twenty six GPs were interviewed (*♂n = 8, ♀n = 18)*. On average the interviews took 54 minutes (range 24 mins – 84 mins). They were conducted at a time and location determined by the participant and no one else was present. The characteristics of the sample and its related population appear in Table 1. According to the ACT Medical Registration Board data, our sample was skewed towards women and those trained in Australia.

**Table 1:** **Characteristics of the sample and comparison with 2007 ACT Medical Registration Board data for GPs working 25 hours or less a week**

	Sample	ACT Medical Registration Board 2007 Part time GPs
	n = 27	n = 56
**Average age (yrs)**		
**Females**	47	45
**Males**	58	61
**Gender (%)**		
**Females**	66	53
**Males**	33	37
**Registration (%)**		
**Vocationally registered**	82	83
RACGP* registrar	4	1
**Other**	14	6
**Unknown**	-	6
**Original training (%)**		
**Australia**	93	72
**Overseas**	7	22
**Unknown**	-	4

*Royal Australian College of General Practitioners.

### Nature and extent of GPs’ paid and unpaid work

Considering all their paid work, the majority of participants (n = 17) were in full-time paid employment, that is, at least 40 hours a week (see Table 2). Only four GPs did not work in another paid position; of these, two were the primary carers of their children and the other two were semi-retired.

**Table 2:** **Gender and number of the sample in paid employment**

Gender	Full-time	Half-time	Less than half-time
**Female**	**12**	**4**	**2**
**Male**	**5**	**1**	**2**

All the GPs in paid employment other than general practice were working in health-related areas, including education and training, policy, research and academia, and medical subspecialties (see Table 3). Importantly, our participants uniformly considered themselves GPs even when engaged in non-clinical work or subspecialties, because they were using their general practice skills and knowledge.

**Table 3:** **Areas of work undertaken by sessional clinical GPs in Canberra**

Area	Example
**Education**	Medical student teaching
	GP vocational training
**Policy**	Government departments
	National health organisations
	GP hospital liaison
**Subspecialty areas**	Drug and alcohol
	Family planning
	Surgical theatre assistance
	Maternal and child health
	Indigenous health
	Refugee health
	Mental health
	Forensic health
**Research & academia**	ANU Medical School

### Reasons for sessional general practice

Gender and the pressures of general practice were mentioned by the majority of participants and will be discussed shortly. Smaller numbers also mentioned the desire for variety in their working life. The flexibility of sessional work allowed many of the GPs to use their medical training in a non-clinical setting doing research or working to improve health policy, and in other clinical subspecialties, such as prison work, indigenous health, family planning and dermatology.

*So with the variety, and the flexibility, and the sort of sub-specialisation which happens in this country, I was able to do what I wanted to do. (♀GP25_40s)*

According to these GPs, sessional general practice is sufficiently flexible that it allows them to pursue complementary career goals. All found that “life’s less boring” (♀GP6_50s) and “more clinically sustainable and interesting” (♀GP18_40s) with flexible work practices. Several GPs found working with vulnerable or special needs communities a satisfying complement to mainstream general practice. The GP quoted below, for example, was drawn towards helping an under-serviced population.

*That was the year that the World Health Organization report came out about Aboriginal health, and I thought “That is pretty appalling!” … I discovered there was an Aboriginal health service here, so I went and got a job there and worked there. (♀GP15_40s)*

Gender strongly influenced female participants’ decision to work less than full-time. Thirteen female GPs and one male GP had dependent children, but only the man did not mention his children or family during the interview. Three of the mothers commented that their spouse’s employment required them to work sessionally in order to manage the household and caring responsibilities. A further two women with adult children had significant caring responsibilities. Several male GPs acknowledged that the impact of having children was felt more keenly by women, but only two men mentioned that family commitments played a role in their decision to work sessionally.

Caring responsibilities were universally viewed by the mothers in the sample as personally important and rewarding. They wanted to be involved in parenting and their children’s school activities, and felt it was important to fit work around school hours. None resented or regretted the need for flexible hours around children. One GP even attributed an improvement in her practice skills and knowledge to motherhood.

*I learned an awful lot by having babies. I give much better breast feeding advice after I had babies than before [sic]. (GP♀14_50s)*

Several women stated that the capacity to both train and work less than full-time drew them towards general practice. A few female GPs mentioned that they were increasing or expected to increase their hours as their children became more independent. This was borne out in our sample with the four older women in full-time paid employment, but notably still only working sessionally in general practice.

The pressures associated with general practice were keenly felt by our participants.

*Frankly I find general practice extremely stressful at times and that is one of the reasons I went to teaching. (♀GP13_50s)*

*I had just had enough of the burden of it, I was worn out. (♂GP24_50s)*

*It got tiring and that’s about it really. (♂GP11_50s)*

*The nature of general practice … influenced my decision [to work sessionally] more than anything. Just that it’s very full on and very relentless and [it’s] an onslaught of humanity at times. (♂GP19_30s)*

These quotes refer to the emotional and physical consequences of general practice. The last quote talks about the “nature of general practice” and one could easily argue that this has always been so. However, the majority of our participants felt that something had changed over recent years, in part because the balance of conditions within the consultation had altered. They perceived a shift away from traditional “disassociated problem solving” (♀GP20_40s) involving a mix of semi-acute and chronic care, towards the management of multiple, chronic diseases.

*The nature of the demographic of the patients is such that they’re often older. They’ve got complex medical conditions. You can’t see them in 15 minutes, especially if they’re new. (♀GP3_50s)*

*I think all general practices are finding that we’re seeing more and more complicated work. … I don’t get many of the things that we would say are simpler. Most of the people who are booked up are people with chronic mental health issues from pain, compensable issues, elderly people with complicated diabetics. (♀GP14_50s)*

Many GPs expressed the tiredness that results from this style of work with complex patients.

*I think they [patients and the College] expect that you go and look at it in a more holistic way. I think it is extremely hard to do that full-time. I just think it is hard to maintain that focus. (♀GP27_50s)*

*To attend to a person properly, and to listen to them and make them feel listened to, and to deal with their physical problems, all in fifteen minutes, is tiring. (♀GP26_40s)*

Additionally, the participants felt that their patients’ conditions increasingly required psychological management, which had consequences for the treating doctor.

*It doesn’t matter what the consultation is about, it can still end up turning into some emotional issue… I find that quite exhausting. (♀GP7_40s)*

*General practice is intellectual work all the time, with a huge emotional overload. (♀GP1_40s)*

For the GPs in this study, the prevalence of complex, chronic illness and the increasing need for psychological management meant that consultations were time consuming and exhausting.

*It’s hard to, you know, [appreciate] the complexity of managing multiple health issues in individuals who [you have] known for a long time.... Knowledge of what someone’s done before and why something might impact on them, and I think it’s hard to define how much time and energy goes into that sort of thing. (♀GP14_50s)*

Increasing patient expectations were also perceived to define current general practice. Patients appeared to be more demanding and hold unrealistic expectations.

*Most of my patients … wouldn’t be happy if you just printed out a script and handed it to them … What might happen if you do take antibiotics? What might happen if you don’t take the antibiotics? [What are] the reasons for taking it? [What are] the reasons not for taking it, you know? I think that takes up a lot of time and I think that’s quite exhausting. (♀GP7_40s)*

*On the one hand they are saying you should be home with your babies and on the other hand they want you there 24/7. (♀GP1_40s)*

The GPs we interviewed felt these expectations keenly.

### What sessional general practice offers

Sessional general practice was compared favourably to full-time general practice. The strain of full-time clinical practice strongly influenced many GPs’ work decisions. Sessional clinical practice was seen to offer “downtime” (♀GP18_40s), the opportunity to “recharge your batteries” (♀GP3_50s). It kept them “fresh,” (♂GP11_50s) provided time to “catch your breath” (♂GP19_30s), and allowed GPs to “maintain good mental and physical health” (♀GP14_50s). Therefore, many of the GPs felt that a mix of clinical, non-clinical and unpaid activities attenuated the tiredness one might otherwise feel. Time away from clinical work also gave one GP the opportunity to think about complex clinical cases (♂GP11_50s).

Many of our participants felt that full-time general practice did not allow them to be the best GP they could be.

*[Like] most GPs I want to do a decent job, and I have actually always found that if I go beyond a certain number of sessions I don’t think I am doing a decent job anymore. (♀GP27_50s)*

*I think I’m a better GP by not doing it all day. … By Friday afternoon I’m not really that interested in people’s small petty problems. (♀GP20_40s)*

*If you are doing general practice well clinically, it is quite challenging. I have seen a lot of lazy GP’s that palm things off. (♀GP23_60s)*

Our participants recognized that “inner resources” were central to providing good quality care.

*If you’re part-time you’ve got more [inner] resources to be able to offer that particular type of [patient]. (♀GP18_40s)*

*The level of compassion and I think … the care factor would diminish. … Doing it less than full-time gives you the capacity to, I guess, rebuild yourself, so that you can do your job well. (♀GP12-20s)*

These rhetorical claims were buttressed by their actions. For example, dedication could be expressed through thoroughness, as below.

*I tend to review all the records before I write a complex referral say to a cardiologist or a physician. I will attach lots of information, because… I have a duty to give them all the information, and say “Well the question I really want you to answer is this.” (♂GP17_60s)*

Commitment to patients was also revealed in the way several participants had chosen to spread their sessions across more days to ensure continuity of care, rather than the same hours across working fewer days

*“I needed to have some continuity of care with my patients. … While it would be preferable to just do … two and a half days … it ends up being easier, in terms of seeing people again. … It works better … if you work more days. (♀GP12-20s)*

The remuneration for sessional clinical work was generally seen as modest, particularly due to the number of patients GPs saw with chronic and complex diseases and the associated unpaid paperwork. Continuing medical education was seen as important but several GPs found it slightly more difficult to keep up to date clinically.

## Discussion

Does less than a full-time patient load reflect a lack of commitment among general practitioners? We think not. The majority of the GPs we interviewed were in full-time paid employment overall and when not consulting with patients in general practice they used their knowledge and expertise in other areas of the health system. This finding challenges the perception – sometimes seen in the medical press – that sessional clinical practice is a personal indulgence that disregards the needs of the community [15]. The disadvantages of working less than full-time serves to reinforce our claim that these GPs were committed health professionals.

Gender undoubtedly influenced the decision of the female participants to work sessionally. This is consistent with the international literature, which indicates caring roles and the presence of young children are predictive of less than full-time clinical hours [17–21, 24].

An unexpected finding was that many participants felt clinical work was becoming more taxing because patients tended to be older, with more complex conditions, and with a greater need for psychological care. This perception is confirmed by the quantitative data gathered via a national Australian study of general practice between 2002–03 and 2011–12 [36]. The participants also believed that patients were becoming more demanding and held unrealistic expectations of what could be achieved within a 15 minute consultation. Therefore, the majority of GPs interviewed used sessional work as a way of managing the various personal and professional demands in their life. This common sense approach to managing the changed work conditions that have increased pressure on GPs serves to underscore rather than undermine their commitment to their patients and profession.

Our study was able to establish a link between the increasingly complex and demanding nature of the consultation, and the way sessional work enables GPs to remain in the clinical workforce providing high quality general practice services. Other studies have established a significant link between job satisfaction and hours worked [21, 27], but unlike ours they did not explain the reasoning behind the association or how their work might be creatively managed to attenuate the pressures. Since working fewer hours is associated with greater job satisfaction and less stress [27, 28], and without any loss of patient trust and satisfaction [28] sessional work can be seen as both a rational and a highly professional response to changed working conditions.

Participants ranged in professional experience, worked in an assortment of employment circumstances, included partnered and single GPs, and those with and without children. We contend that the research captures the range of views held by ACT general practitioners working sessionally in clinical practice. GPs at all stages of their career had chosen to work sessionally. This is contrary to qualitative research from the UK, which suggests that the younger generation of GPs “organise their general practice work to suit their own needs,” and that this represents a generational shift. [25] The same authors claim that that quality of work and life has replaced a commitment to patients and the profession. However, our study uncovered no evidence of a decrease in commitment to either patients or the profession. Our participants valued contributing in both clinical and non-clinical settings, and adapted their work types and hours to achieve this.

Sessional general practice was seen to offer a way of maintaining the quality of their services, thereby demonstrating their commitment to patients and professional enthusiasm. A mix of clinical, non-clinical and unpaid activities ensured they had time to recover between.

### Limitations

Our sample includes almost half the population of sessional general practitioners in the ACT, according to the ACT Medical Registration Board survey data. However, the Board’s survey is voluntary and most likely incomplete, so we cannot establish our data’s representativeness. Furthermore, the urban nature of the sample means that our findings cannot be extrapolated to rural and remote areas.

Like all research that involves voluntary participation, some bias may be present. The smallness of the GP community meant that many participants knew or were known to at least one of the research team. However, this was just as likely to enhance as to mitigate the frankness of the interview.

The reasons given by the GPs for working sessionally could conceivably be self-justifications. Nevertheless, “if men [sic] define situations as real, they are real in their consequences” [37]. Therefore, it is helpful to understand their perceptions and acknowledge the repercussions, regardless of their veracity as judged by another.

## Conclusions

Current reports on the trend towards part-time general practice may be misleading. Many GPs who carry less than a full-time clinical load could, for instance, be in full-time paid employment. Our data suggest that GPs who are choosing sessional clinical practice do so in order to maintain their high professional standards. Sessional work enables them to maintain their commitment to patients and the profession, while attenuating the challenges that result from the increasing demands of the consultation. Sessional work is, in fact, a rational response to an increasingly difficult work environment. This paper clearly documents the professional commitment and breadth of contributions made by sessional general practitioners, which is often undermined in the medical press [15, 16] and overlooked by current means of collecting workforce data [14].

Our study was initiated in the hope that an understanding of the motivations and challenges of sessional general practice would provide potential levers for policy makers to increase the number of sessions currently work by sessional GPs. However, we found that most were happy with their portfolio of clinical and non-clinical contributions, and many were in full-time paid employment across a range of roles. Instead of encouraging sessional GPs to work more sessions, policy should reframe sessional general practice as an opportunity, rather than a loss. Supporting GPs to develop portfolio careers, which are sustainable, deliver high quality patient care, and contribute in clinical and non-clinical areas, will be more fruitful than wishing for a return to long clinical hours.

## Abbreviations

GP: General practitioners
ACT: Australian Capital Territory
ANU: Australian National University.
